# Novel Nucleus-Oriented Quenched Activity-Based Probes Link Cathepsin Nuclear Localization with Mitosis

**DOI:** 10.1021/acssensors.4c03217

**Published:** 2025-02-17

**Authors:** Karin Reut Shannon, Tommy Weiss-Sadan, Emmanuelle Merquiol, Gourab Dey, Tamar Gilon, Boris Turk, Galia Blum

**Affiliations:** † The Institute for Drug Research, The School of Pharmacy, The Faculty of Medicine, The Hebrew University, POB 12271, Jerusalem 9112001, Israel; ‡ Azrieli College of Engineering, 26 Yaakov Shreibom Street, Jerusalem 9103501, Israel; § Department of Biochemistry and Molecular Biology, J. Stefan Institute, Jamova 39, SI-1000 Ljubljana, Slovenia; ∥ Faculty of Chemistry and Chemical Technology, University of Ljubljana, Večna Pot 113, SI-1000 Ljubljana, Slovenia; ⊥ The Wohl Institute for Translational Medicine, Hadassah Hospital, Kalman Ya’akov Man Street , Jerusalem 9112001, Israel

**Keywords:** quenched-activity-based-probes, nuclear cathepsin, mitosis, cell-cycle, imaging probes, cell-penetrating peptides

## Abstract

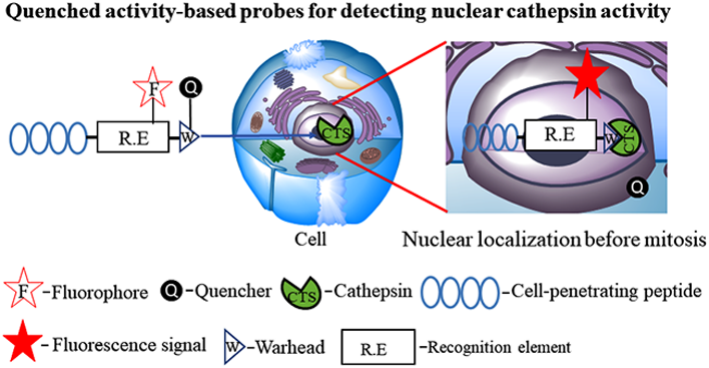

Cysteine cathepsins are important proteases that are highly upregulated in cancers and other diseases. While their reported location is mostly endolysosomal, some evidence shows their nuclear localization and involvement in the cell cycle. We aim to generate tools to investigate the involvement of cathepsins in the cell cycle progression. To investigate nuclear cathepsin activity, we designed nucleus-directed quenched activity-based probes (qABPs) by attaching cell-penetrating peptides (CPPs). qABPs are active-site-directed compounds that enable direct real-time monitoring of enzyme activity by the covalent linkage between the probe and the enzyme’s active site. Biochemical evaluation of the CPP-qABPs showed potent and selective probes; cell fractionation, multimodal flow cytometry-imaging, and time-lapse movies demonstrated nuclear cathepsin activity in living cells. Interestingly, these probes reveal a spatiotemporal pattern, a surge of nuclear cathepsin just before mitosis, suggesting yet unrevealed roles of cathepsin in cell division. In summary, these nuclear-directed qABPs serve as unique scientific tools to unlock the hidden features of cysteine proteases and to understand their involvement in cell division and cancer.

Cysteine cathepsins are proteases implicated in numerous physiological processes that maintain cellular homeostasis. Cathepsins are expressed in several human tissues and have been found mainly in the endolysosomal compartments of the cell, where they are involved in critical activities such as protein turnover, autophagy, and antigen processing.^1,2^ Moreover, cathepsins are also implicated in extracellular matrix (ECM) degradation, especially in pathological conditions such as cancer and atherosclerosis.^3−6^

Over the last years, researchers have found cysteine cathepsin localized in the nucleus, suggesting novel roles for cysteine cathepsins in the cell cycle.^7−9^ Specifically, cathepsin L has been found to cleave nuclear factors such as the transcription factor CDP/Cux,^10−13^ topoisomerase II,^14^ and histone H3.^15^ Duncan et al. showed that nuclear cathepsin L processes histone H3 tail during mouse embryonic stem cell differentiation which may be regulated by acetylation present on the histone tail.^15^ Goulet et al. demonstrated that catalytically active cathepsin L variants localize to the nucleus and regulate cell-cycle progression through proteolytic processing of both the CDP/Cux transcription factor and the N-terminus of the histone H3 tail. In that report, the nuclear Cathepsin L lacked a signal peptide due to different translation initiation, yet it was capable of proper folding and activity.^10^ In addition, cathepsin V, a human variant of cathepsin L that is lacking in mice, was also found to be localized to the nucleus and to bind DNA.^16−18^

Accumulating evidence also suggests that the specific functions of the cysteine cathepsins during cell division might be attributed to their localization at specific time points during the cell cycle.^2,10,19,20^ To this extent, cathepsin L localized in the nucleus was shown to promote the cell cycle in HCT116 cells, its inhibition decelerated the cell cycle, and its expression led to accelerated entry to the S phase.^7^ In addition, a cathepsin L-like protease was found to colocalize with α-tubulin in cells harvested at mitosis, also suggesting new functions for this protease in cell replication.^21^ Lastly, variations in cathepsin-like protease localization were reported; the nuclear-localized enzyme had a function in the cycle; it was found at the spindle during mitosis and in the nucleus after the first cell division. In nonproliferative phases, the cathepsin-like protease was found to be in the cytoplasm.^22^

But how are cathepsins trafficking to the nucleus? The majority of cathepsins are trafficked to the lysosome from the Golgi apparatus after entering the ER, relying on an ER import signal. The nuclear localization of cathepsins has been suggested to arise from different isoforms derived by alternative translation start sites from leaky scanning of the translation initiation, producing transcripts that lack signal secretory peptide sequences.^9,23^ However, Tholen et al. show that out-of-frame AUGs prevent translation of truncated cathepsin L, and therefore, the nuclear localization of cathepsin L is most likely not generated by leaky scanning of its mRNA.^24^ Thus, the means for nuclear localization of cathepsin L are still under investigation.

While these reports highlight nuclear localization and activity of cathepsins, further research is necessary to elucidate the multifaceted role of cysteine cathepsins in the cell-cycle process. An established technique to track and study cathepsins and other proteases is by labeling these proteases with activity-based probes (ABPs). ABPs are small molecules that selectively bind and covalently attach to active proteases. Most ABPs have three components: (1) a short peptide sequence selective toward a target protease. (2) An electrophile (termed “warhead”) that enables covalent modification of the protease active site and increased selectivity. (3) A tag that allows for the detection of the probe-enzyme complex once the protease has been covalently labeled.^25,26^ Quenched ABPs (qABPs) are a version of fluorescently labeled ABPs, which only generate the signal once the probe has bound to the target protease, thereby releasing the quencher, leading to fluorescence and allowing for a specific fluorescent signal on the site of activation in real-time. Although a variety of efficient qABPs specific for cysteine cathepsins have been published, these molecules have reduced cell permeability due to a bulky quencher group, resulting in low cellular uptake and a weaker signal compared with nonquenched probes.^27^

While the qABP technology may enable the investigation of temporal nuclear cathepsin activity, there is a great need for probes with improved cell permeability and nuclear targeting. To overcome these limitations, we designed qABPs with a cell-penetrating peptide (CPP), a poly-positive moiety that delivers bioactive substances into the cells by penetrating through the cell membrane. A highly used CPP is the trans-activator of transcription (TAT peptide), which consists of multiple positively charged amino acids.^28^ Often, cargos are covalently coupled to CPPs; e.g., by fusion proteins or chemically prepared conjugates.^29^ Interestingly, TAT-derived peptides are not only capable of penetrating the cell membrane but can also lead to nuclear entry, binding to nuclear components, and importing large nanoparticles.^30^ Nuclear localization sequences (NLS) are used for similar purposes; they penetrate the nuclear membrane barrier and promote nuclear translocation.^31,32^ NLS are composed of a short sequence of positively charged amino acids, which are recognized by nuclear transport proteins (e.g., importin-α and importin-β).^33^

In this study, we directed our potent cathepsin ABPs to the cell and to the nucleus by attaching CPP moieties to the probe’s peptide sequence, generating a new library of CPP-ABPs.^27^ We show detailed synthesis and chemical evaluation of the library and efficient labeling of cathepsins B and L in vitro, in addition to evaluating the potency and cell permeability. Most importantly, using real-time high-resolution fluorescent microscopy, we were able to reveal a correlation between cell mitosis and cathepsin nuclear localization.

## Methods

All solvents were HPLC-grade. The peptides were prepared using standard solid-phase peptide synthesis. All water-sensitive reactions were performed in anhydrous solvents under a positive pressure of argon. All light-sensitive reactions (in the presence of the quencher or fluorophore) were performed in the dark. Reactions were monitored and evaluated by LC-MS reversed phase with C18 or C4 columns using a gradient of water/acetonitrile supplemented with 0.1% formic acid. Reverse-phase preparative HPLC was used for separations and purifications with C18 or C4 columns with a 0.1% trifluoroacetic acid in water/acetonitrile gradients. The final molecules were analyzed by high-resolution MS, MALDI-ToF. Fluorescent gels were scanned using a Typhoon FLA flatbed laser scanner (GE Healthcare). Probe names, KRS, are the initials of the lead author who synthesized them.

### Synthesis of 2,6-Dimethyltherephthalic acid (**1**)

**Scheme 1:**
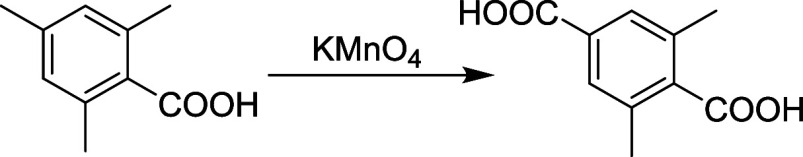
Synthesis of **1**, 2,6-Dimethylterephthalic Acid from 2,4,6-Trimethylbenzoic Acid

Dimethyltherephtalic acid (DMTA) was prepared from 2,4,6-Trimethylbenzoic acid (1 equiv) dissolved in NaOH 1 M by oxidation with KMnO_4_ (2 equiv) for 2 h (Scheme 1). The reaction was stopped by adding 50% w/v H_2_SO_4_ and then saturated NaHSO_3_ was added. The product was extracted using ethyl acetate and then dried yielding a beige color powder.

### Bromomethyl Ketone (BMK) Synthesis (**2**)

The following method was adapted from a previous protocol.^27^ BMK was prepared from Lys-(Boc)–OH. To a solution of amino acid (3.38 g, 12.4 mmol) and *N*-methylmorpholine (1.34 mL, 12.4 mmol) in anhydrous THF (124 mL) at −20 °C was added isobutyl chloroformate (1.58 mL, 12.4 mmol). The reaction mixture was stirred for 15 min. Diazomethane, prepared from Diazald (7.25 g, 34.3 mmol), was added slowly to the reaction mixture while maintaining the temperature at −20 °C. The reaction mixture was treated with a solution of (40% aqueous HBr: acetic acid) (1:1) (1.03 mL) and stirred for 15 min at 0 °C. The reaction mixture was diluted with EtOAc (150 mL) and washed with water, then with aqueous saturated with NaHCO_3_ (2 × 50 mL), and aqueous saturated NaCl (50 mL). The organic layer was dried with MgSO_4_, filtered, and concentrated under reduced pressure to form a yellow oil (85% yield). The crude material was used for the subsequent reaction.

### Phe-Phe-Maleimidopropionic Acid Synthesis (**3**)

Fmoc-Phe (0.75 mmol) was loaded onto 2-Chlorotrityl resin (0.5 mmol), (Sigma-Aldrich), by shaking with 3 equiv of DIEA in dry DMF for 1 h; then methanol was added to quench the unreacted resin. The resin load was quantified. The Fmoc group was deprotected using 20% Piperidine in dry DMF for 20 min twice as described in ref (27). Then, 3 equiv of Fmoc-protected Phe was coupled with HOBT (3 equiv) and DIC (3 equiv) for 2 h. The resin was washed with DMF and DCM. Fmoc was deprotected as described above, and 3 equiv of maleimidopropionic acid (Sigma) was coupled with HOBT (3 equiv) and DIC (3 equiv) in dry DMF for 2h. The peptide was cleaved from the resin by the addition of 2% TFA/DCM. The combined fractions were evaporated with toluene, and the crude peptide was lyophilized to yield 94% which was used for further syntheses.

### Synthesis of the CPP-qABP Scaffold (**4**)

**Scheme 2:**
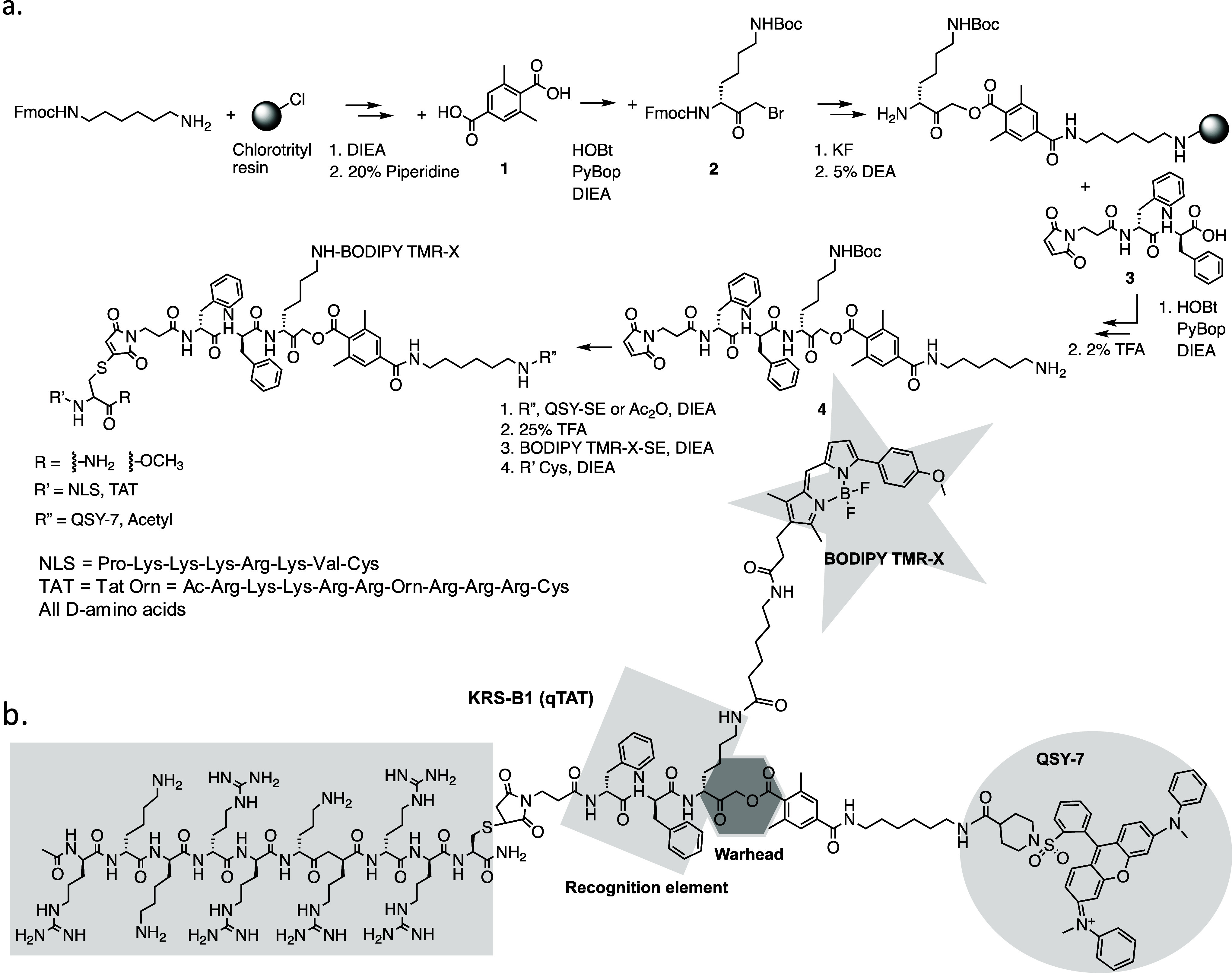
Synthetic Route of Compound **4** (**KRS-B1**)

CPP-qABPs were prepared by loading 2-Chlorotrityl while shaking with Fmoc-1,6 diaminohexane hydrochloride and 3 equiv of DIEA in dry DMF for 1 h, and then methanol was added to quench the resin. The resin load was quantified. Fmoc was deprotected as previously described, and 3 equiv (relative to the resin load) of **1** (which was first preactivated with 1.5 equiv HOBt, 1.7 equiv PyBop, and 6 equiv DIEA in DMF to form an ester) was added. The reaction mixture was shaken for 2 h. The resin was then washed with DMF and DCM. Three equiv of **2** that was premade as described above and 10 equiv of KF potassium fluoride were added to the free amine for 2 h to generate the AOMK. The Lys Fmoc deprotection was performed using 5% DEA in DMF for 20 min, then quickly washed, and coupled to 3 equiv of the previously synthesized, **3**. The protected peptide was cleaved from resin by the addition of 2% TFA/DCM. The combined fractions were co-evaporated with toluene, and the crude was HPLC-purified using preparatory reverse phase C4 column, water–acetonitrile gradient 0.1% TFA). The product was eluted at 37% acetonitrile followed by lyophilization to yield compound **4** (Scheme 2) as a white-yellow solid (1.5% yield).

### Coupling of the Quencher QSY-7 to **4**

A 1.2 equiv of QSY-7 succinimidyl ester (Invitrogen) in DMF and 9 equiv DIEA were added to purified **4** for 5 h. The reaction was monitored every 30 min by LC-MS. Hexanediamine-DMTA-Lys (Boc)-Phe-Phe-maleimidopropionamide-QSY-7 (**4-QSY-7)** was purified by a C4 column and eluted by 60% acetonitrile, and the compounds were lyophilized to obtain a dark purple powder (79.6% yield).

### Coupling of Fluorophore BODIPY TMR-X to **4-QSY-7** Generating a qABP

The Boc protecting group was removed from the lysing of **4-QSY-7** using 25% TFA in DCM for 30 min, and the reaction was monitored by LC-MS. BODIPY TMR-X (Invitrogen) was coupled (similar to the quencher coupling described above) with an average of 27% yield to obtain a dark purple powder.

### Coupling of TAT-Derived Peptide to qABP Generating KRS-B1(qTAT)

The TAT peptide (Arg-Lys-Lys-Arg-Arg-Orn-Arg-Arg-Arg-Cys, all D-amino acids, except for the Cysteine) was custom synthesized by the Bio Basic Canada Inc. TAT was coupled via its C-terminal Cysteine thiol to the maleimide of the peptide in DMF (100 mM final concentration, 3 equiv) and 9 equiv DIEA. The coupling reaction was monitored for 6 h, purified using a C4 column, and eluted at 63–65% acetonitrile. After the lyophilization, the product was obtained as a dark purple powder, with a 0.7% yield.

### Coupling of NLS Peptide to qABP Generating KRS-E1(qNLS)

The NLS peptide Pro-Lys-Lys-Lys-Arg-Lys-Val-Cys was custom synthesized by Bio Basic Canada Inc. NLS coupling was performed similarly to the TAT coupling as described above. The purification was performed using C4 column, and products were eluted at 50–100% acetonitrile with 1.0% yield.

### Synthesis of Acyl Containing ABPs

The probes KRS-D1(qAc) and KRS-C1(Ac) were prepared similarly to KRS-B1(qTAT) and KRS-A1(TAT), respectively, except an acetylated cysteine was used instead of the TAT. These molecules served as control compounds.

### Synthesis of Nonquenched ABPs KRS-A1(TAT), KRS-C1(Ac), KRS-F1(NLS)

These compounds were synthesized by adding **4** to acetic anhydride 10 equiv and DIEA 15 equiv dissolved in anhydrous DCM. The reaction was shaken for 20 min to receive nonquenched compounds. The coupling of BODIPY TMR-X and then TAT, acetylated cysteine, or NLS was done as described above.

## Biochemical Evaluation

### Quenching Efficiency

Increasing concentrations (0.013–10 μM) of KRS (A1-F1), in acetate buffer (50 mM acetate, 5 mM MgCl_2_, 2 mM DTT, pH 5.5), were prepared in a 96-well plate. Fluorescence was measured and quantified using a BioRad plate reader, Ex./Em. 535/570 nm. Arbitrary fluorescent units were plotted versus probe concentration, and the ratio between the slope of each quenched probe and the average of the nonquenched probes were determined as quenching efficiency.

### Recombinant Cathepsin Labeling

Recombinant human cathepsins B (0.7 μg), L (0.6 μg), or S (0.7 μg) in reaction buffer (50 mM acetate, 5 mM MgCl_2_, 2 mM DTT, pH 5.5) was pretreated with the cathepsin inhibitor **GB111-NH**_**2**_ or vehicle for 30 min at room temp. Indicated concentrations of KRS probes were added to the samples for 30 min at 37 °C. The reaction was stopped by adding 4× sample buffer (40% glycerol, 0.2 M Tris/HCl 6.8, 20% β-mercaptoethanol, 12% SDS, and 0.4 mg/mL bromophenol blue). The samples were boiled and separated by a 12% SDS gel and scanned by a Typhoon laser scanner at Ex./Em. 535/570 nm for fluorescence.

### Cell Culture

NIH-3T3 mouse fibroblast cells or HeLa cells human epithelial cells of uterine cervix adenocarcinoma were cultured in DMEM (Dulbecco’s modified eagle’s medium) supplemented with 10% fetal bovine serum (FBS), 1% penicillin, and 1% streptomycin in a humidified atmosphere of 95% air and 5% CO_2_ at 37 °C.

### Evaluation of Probe Labeling in Intact Cells

NIH-3T3 cells (2.5 × 10^5^ cells/well) were seeded in a 12-well plate 1 day prior to treatment. Cells were pretreated with inhibitor **GB111-NH**_**2**_ or vehicle for 30 min followed by incubation with **KRS** probes at indicated concentrations predissolved in 0.1% DMSO and 0.9% ethanol in a culture medium. After 6 h of probe incubation cells were washed with PBS and lysed by addition of sample buffer as described above. Lysates were boiled for 5 min, centrifuged, and separated by 12.5% SDS-PAGE. Cathepsins labeled in cells were visualized by scanning the gel with a Typhoon laser scanner at Ex./Em. 535/570 nm.

### Evaluation of Probes Stability in Intact Cells-Time Course

NIH-3T3 cells (2.5 × 10^5^ cells/well) or HeLa cells (2.0 × 10^5^ cells/well) were seeded in a 12-well plate 1 day before treatment. Cells were incubated with KRS probes at 2.5 μM predissolved in 0.1% DMSO and 0.9% ethanol in a culture medium for indicated durations. After the incubation, cells were washed with PBS and lysed by the addition of sample buffer. Samples were analyzed by fluorescent SDS-PAGE as described above.

### Live Cell Imaging

NIH-3T3 or HeLa cells were seeded in an 8-well coverslip chamber (Lab-Tek) at a density of 100,000 cells per well and grown at 37 °C under 5% of CO_2_, 24 h later, cells were treated with 2.5 μM **KRSs** probes for 6 h. The medium was replaced with DMEM without phenol red, and Hoechst was added to a final concentration of 5 μg/mL. Live imaging of cells using a Zeiss LSM 710 Axio Observer.Z1 with a 63/24 Oil DIC M27 lens, in Cy3 and DAPI channels, was performed over 24 h; pictures were taken every 20–25 min. The Z-stack analysis was performed using 3–5 different depths focusing on the cell nucleus; each slice was 1–1.5 μm. Image processing was performed using NIS-Elements image acquisition software, quantifying the 63× images of several experiments and calculating the average fluorescence intensity. Statistics and standard deviation were calculated by using Microsoft Excel.

### Fractionation

Cell fractionation was performed according to the manufacturer’s instructions using the Subcellular Protein Fractionation Kit for Cultured Cells (Pierce, Thermo Fisher Scientific Inc., Rockford, IL, USA, number 78840). Lysed cells were separated into subsequent cytosol, membrane, and nuclear fractions. Extracted proteins were subjected to SDS PAGE separation and fluorescence and Western blot analysis.

### Western Botting

After separating the protein mixture by SDS-PAGE, the gel was transferred to PVDF membranes using an electric field. Blocking was performed with 5% BSA diluted in Tris-buffered saline- 20% tween 20 (TBST) for 1 h at room temperature. After 3 washes with TBST, 5 min each, membranes were incubated with the following primary antibodies: Cat B (1:1000, Santa Cruz Biotechnology, Dallas, Texas, USA SC6046), Cat L (1:2000, R&D Systems, Minneapolis, MN, USA BAF952), PARP (1:1000, Cell Signaling Technology, Beverly, MA, USA, CS9542) and tubulin (1:1000, Abcam, Cambridge, UK, ab6046)

Second antibodies: Rabbit anti-Goat IgG-HRP conjugate (1:5000, Bio-Rad Laboratories, Hercules, CA, USA, 1721034). Streptavidin poly-HRP (1:5000, Thermo Fischer Scientific, San Jose, CA, USA, 21140). The signal was detected using the appropriate HRP-conjugated secondary antibody/Streptavidin, followed by an ECL assay (Biological Industries, Kibbutz Beit Haemek, Israel). Visualization of the chemiluminescent protein bands was performed using a Bio-Rad ChemiDoc XRS chemiluminescence detection system.

### Image Streamer

Live or methanol fixed cells were pretreated with KRS probes at 2.5 μM for 6 h predissolved in 0.1% DMSO and 0.9% ethanol in a culture medium. After the incubation, cells were washed with PBS centrifuged and dissolved in PBS in a final volume of 50 μL. The DNA markers: Hoechst (Thermo Fisher) at a final concentration of 1 μg/mL or Draq5 (Thermo Fisher) at a final concentration of 1.5 μM were added. The samples were analyzed using an Image StreamX Flow Imager system (Merk Millipore).

## Results

### Development of Nuclear-Penetrating qABPs and Their In Vitro Evaluation

To investigate cathepsin function in the nucleus, we generated a library of selective cathepsin ABPs that included both quenched and nonquenched ABPs, attached to CPPs. These probes were generated as a modification of the basic qABP design that was previously reported for targeted cell-based imaging of cysteine cathepsin activity.^34^ When an active cysteine cathepsin binds to the probe, the covalent reaction occurs via the electrophilic moiety acyloxymethyl ketone (AOMK). The quencher is then released and the enzyme-bound probe becomes fluorescent reporting on the activity and localization of the enzyme, see Scheme S1 in Supporting Information.

The synthesis of nuclear cathepsin-targeted qABPs was based on the synthetic route of **GB137**, a qABP described by Blum et al.^34^ using the solution and solid phase peptide synthesis (SPPS), with several modifications (see outline in Scheme 2a). The structure of a TAT containing qABP (KRS-B1(qTAT)) is brought as an example of a CPP-qABP that was generated in Scheme 2b.

#### Probe Design

In this probe library, the original carboxybenzyl (Cbz) group in **GB137** was replaced with the phenylalanine amino acid (Phe) at the P3 position to allow for attachment of a “chemical handle” at the P4 position. A maleimidopropionamide was placed at P4 to enable attachment to a cysteine-containing CPP, either cysteine TAT or cysteine NLS peptide, through maleimide–thiol coupling (Scheme 2a). The fluorophore-quencher pair selected was BODIPY TMR-X and QSY7, which enables optimal quenching, as there is a direct overlap between the BODIPY emission and the QSY7 absorption. The BODIPY TMR-X is a cell-permeable fluorescent molecule, thus enabling high-resolution live fluorescent microscopy in cells using the probes. Controls lacking a CPP (capped with an acetyl group) were generated to evaluate the contribution of the two CPPs to cell permeability and nuclear localization. To generate nonquenched ABPs, an acetyl group was placed instead of the quencher moiety, leading to close analogs that differ only in the quencher moiety. The AOMK was selected as the warhead because of its high specificity to cysteine proteases; (Table 1) for the description of probes.

**Table 1:** Description of the Probes

name	probe description	*M*_w_	% yield
KRS-A1 (TAT)	TAT-Cys-Phe-Phe-Lys-(TMR-X)-AOMK-Ac	2882	4.2
KRS-B1 (qTAT)	TAT-Cys-Phe-Phe-Lys-(TMR-X)-AOMK-QSY7	3391	0.7
KRS-C1 (Ac)	Ac-Cys-Phe-Phe-Lys-(TMR-X)-AOMK-Ac	1592	3.1
KRS-D1 (qAC)	Ac-Cys-Phe-Phe-Lys-(TMR-X)-AOMK-QSY7	2190	1.5
KRS-E1 (qNLS)	NLS-Cys-Phe-Phe-Lys-(TMR-X)-AOMK-QSY7	2998	1.0
KRS-F1 (NLS)	NLS-Cys-Phe-Phe-Lys-(TMR-X)-AOMK-Ac	2400	3.7

After completion of the synthesis, the compounds were purified by preparative HPLC and characterized using LCMS and high-resolution mass spectrometry; the detailed synthesis is described in the Methods section. The overall yields of the final products varied between 0.7–4.2%. In total six molecules were generated, two conjugated to TAT, KRS-A1(TAT) and KRS-B1(qTAT), two conjugated to an acyl group KRS-C1(Ac) and KRS-D1(qAc), and two conjugated to NLS KRS-F1(NLS) and KRS-E1(qNLS). KRS-A1(TAT), KRS-C1(Ac), and KRS-F1(NLS) are nonquenched and KRS-B1(qTAT), KRS-D1(qAc), and KRS-E1(qNLS) are quenched probes (Table 1).

### KRS Probes Are Highly Quenched

**Figure 1:**
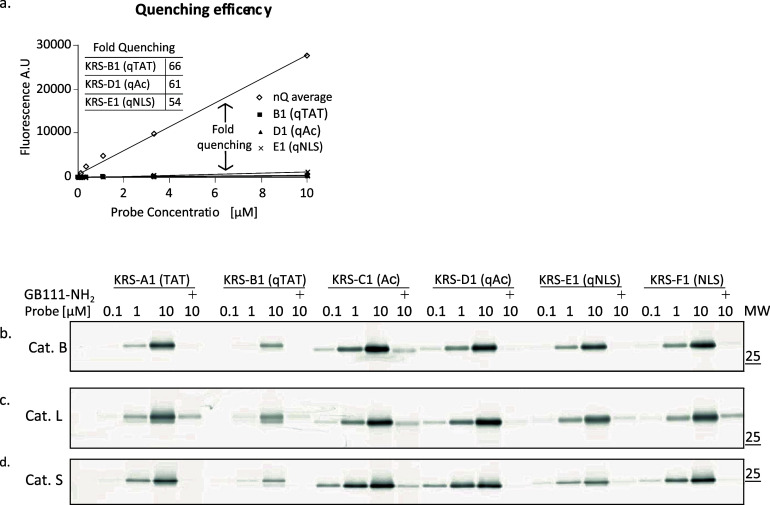
Probe quenching efficiency and binding with recombinant cathepsins. (a) Quenching efficiency. Increasing concentrations of KRS probes in acetate buffer were measured for TMR-X fluorescence. Fold quenching of KRS probes B1, D1, and E1 was calculated by the slope ratio of each probe relative to the average of all nonquenched probes, see Table 1. (b–d) Direct labeling of recombinant cathepsins with KRS probes, proteases were pretreated with cathepsin inhibitor **GB111-NH**_**2**_ or with DMSO vehicle followed by labeling with probes at the indicated concentration for 1 h. Samples were analyzed by fluorescent SDS-PAGE, and bands show fluorescently labeled protease. (b) cathepsin B, (c) cathepsin L, and (d) cathepsin S. This experiment was repeated three times with similar results.

The quenching efficiency of the molecules was measured using a fluorescent plate reader. The fluorescence intensity of each quenched probe was plotted relative to its concentration in comparison to the average of all the nonquenched probes. Quenching efficiency was calculated as the ratio between slopes of quenched and nonquenched fluorescence (Figure 1a). KRS-B1(qTAT), KRS-D1(qAc), and KRS-E1(qNLS) were found highly quenched with 66-, 61-, and 54-fold lower fluorescence than the nonquenched probes, respectively. Although KRS-E1(qNLS) had the lowest quenching efficiency, it was still completely suitable for our purposes.

### Biochemical Evaluation of KRS Derivatives

#### KRS Probes Directly Label Recombinant Cathepsins

Next, we evaluated the affinity of recombinant human cysteine cathepsins B, L, and S to KRS probes. Enzyme-probe interaction was detected on SDS- PAGE, by measuring the fluorescence signal of the bound probe. As a control, the samples were pretreated with a cathepsin inhibitor (**GB111-NH**_**2**_^27^) to show activity-dependent labeling. This in vitro assay demonstrated that all KRS probes were able to bind to recombinant cathepsins in an activity- and dose-dependent manner (Figure 1b–d). Furthermore, the fluorescence signal was dramatically reduced when the enzymes were treated with a competitor compound, **GB111-NH**_**2**_. Thus, we conclude that our probes are specific and commensurate with cathepsin activity.

Notably, the intensities of KRS-B1(qTAT) and KRS-E1(qNLS) labeling were slightly lower than the intensities of KRS-D1(qAc) labeling. The signal decrease in probes containing CPP moieties suggests that large probes such as KRS-B1(qTAT) and KRS-E1(qNLS) are sterically hindered, reducing their entry into the catalytic pocket of the enzyme.

#### KRS Probes Label Endogenous Cathepsin in Intact Cells

Next, we examined whether these probes could label endogenous cathepsins in live cells and whether CPP conjugation improves cathepsin labeling. Monolayers of live NIH-3T3 were pretreated with the cathepsin inhibitor, **GB111-NH**_**2**_, or DMSO vehicle, and then labeled with KRS probes for 6 h by simply adding the probes to the growth media. Cells were then lysed, separated by SDS-PAGE, and visualized for Cy3 fluorescence by a Typhoon scanner.

**Figure 2:**
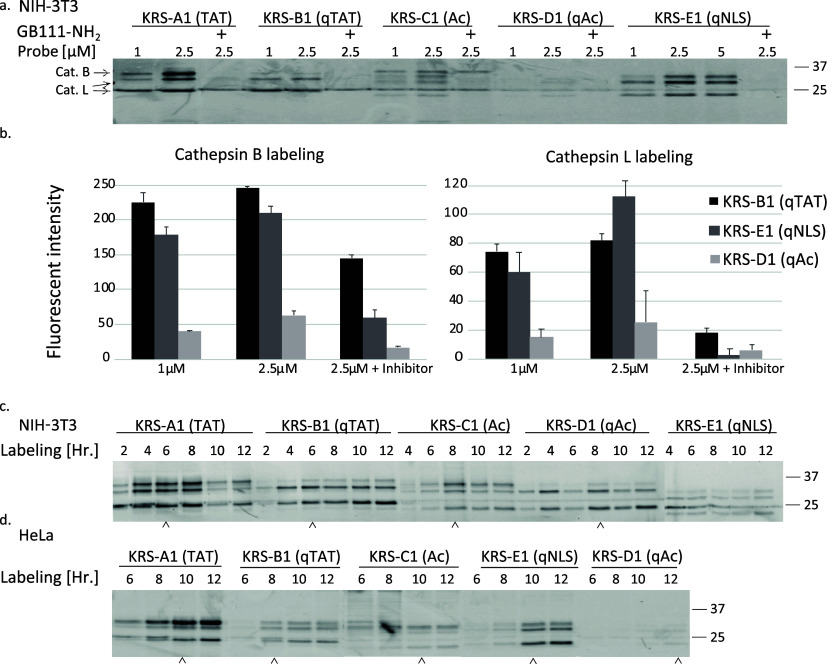
Labeling of intact cathepsins in cells. (a) Intact monolayers of NIH-3T3 were pretreated with the **GB111-NH**_**2**_ or with DMSO vehicle followed by 6 h labeling with probes at the indicated concentration. Cells were collected, lysed, separated by SDS-PAGE and analyzed for Cy3 fluorescent by a Typhoon laser scanner. This experiment was repeated more than three times with similar results. (b) Plots show the quantification average of band intensity of cathepsin B labeling (left) and cathepsin L labeling (right), using Image-J software. Labeling of intact cells over time as in a. using 2.5 μM probe in NIH-3T3 cells (c) and HeLa cells (d) arrowhead indicates optimal labeling time. * Compound F was not applied to kinetic labeling.

We found that KRS probes freely penetrated the cells and labeled endogenous cathepsins in a dose-dependent manner (Figure 2a). The decrease in labeling with samples pretreated with inhibitor (**GB111-NH**_**2**_) indicates that the binding is highly selective for the cathepsins and is dependent on their activity. We detected a 2-kDa shift of the protease-labeled bands with CPP-containing probes as expected from the large probe size. Importantly, probes containing CPP (KRS-A1(TAT), KRS-B1(qTAT), and KRS-E1(qNLS) showed enhanced labeling of both intracellular cathepsin B and L in comparison to probes without the CPP as seen in the intensity analysis (Figure 2). Quantification of all probes is shown in (Figure S1). Noteworthy, the CPP-containing probes have higher cathepsin B and L labeling despite their lower affinity for the recombinant enzymes, demonstrating that the CPP group indeed enhanced the cell permeability of the probes. Moreover, the optimal concentration of the probes was found to be 2.5 μM which was used in the subsequent experiments (additional concentration calibration was done, not shown). In addition, wild-type mouse embryonic fibroblasts (MEFs) and knockout cells were used to confirm that the identity of the labeled enzymes is indeed cathepsin B and cathepsin L (Figure S2).

To determine the optimal duration for labeling with the KRS probes, kinetic experiments were performed in both NIH-3T3 (mouse) and HeLa (human) cells. We found that extended labeling times increased the intensity of the probe signal. For NIH-3T3 the optimal probe incubation times were found to be 6–8 h for most probes (Figure 2c), while HeLa cells needed 10–12 h (Figure 2d) for optimal signals, respectively. Similar to before, CPP-containing probes showed enhanced and accelerated permeability enabling intensified labeling, as demonstrated for the nonquenched (KRS-A1(TAT) in comparison to KRS-C1(AC) and the quenched probe (KRS-B1(qTAT) in comparison to KRS-D1(qAC) in NIH 3T3 and HeLa cells.

#### CPP Probes Are Cell-Permeable and Label Nuclear Cathepsin(s)

**Figure 3:**
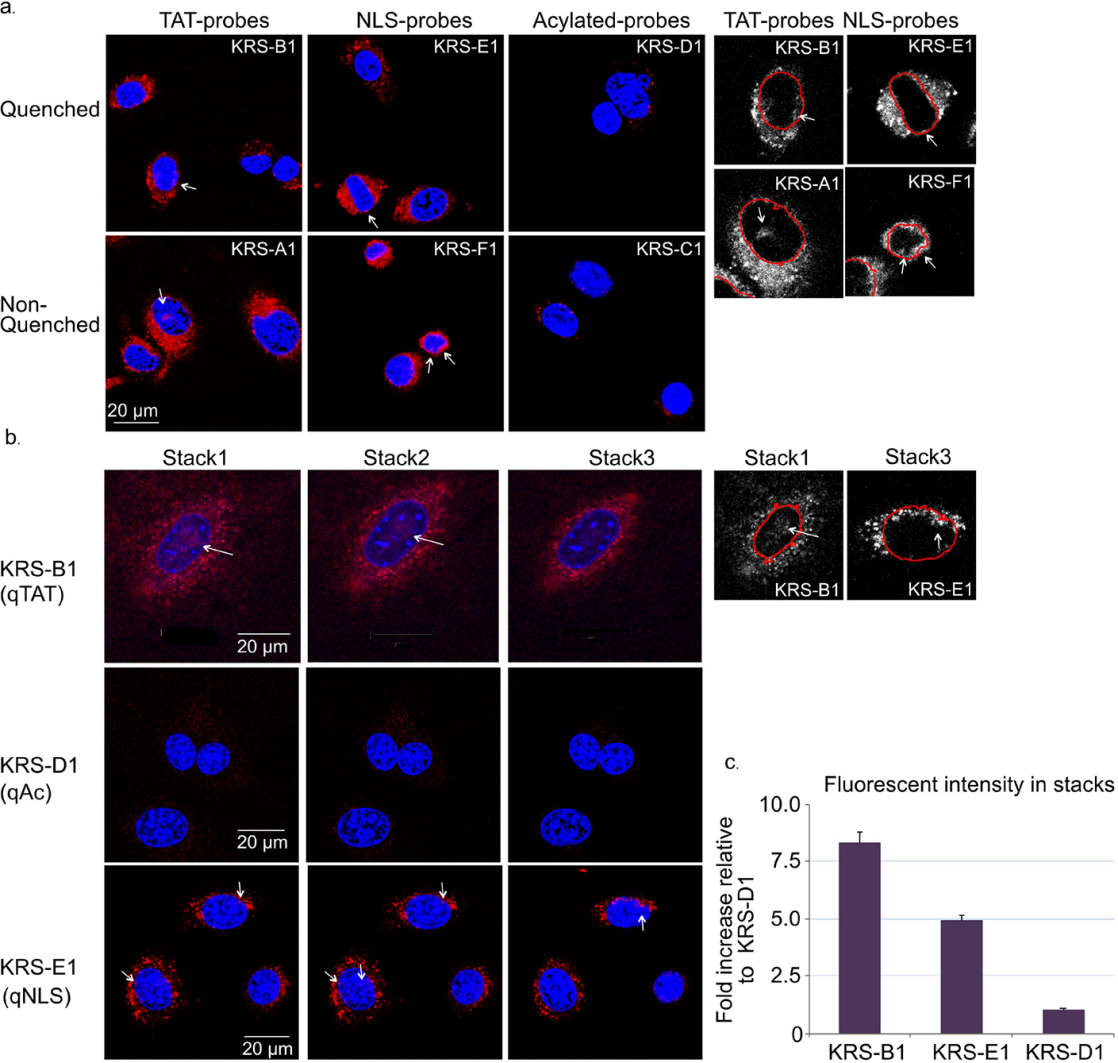
KRS Probe cell permeability and location in cells. (a) Intact monolayers of NIH-3T3 were labeled with 2.5 μM probes KRS probes (red) for 6 h. nonquenched probes were washed twice followed by Hoechst stain (blue), and images were captured with a confocal microscope. The white arrow indicates a nuclear cathepsin stain. On the right, we present enlarged black-and-white images of the probe’s fluorescence with the nucleus contour marked in red (only selected cells with arrows are shown). (b) Intact monolayers of NIH-3T3 were labeled with 2.5 μM quenched probes KRS-B1(qTAT), KRS -E1(qNLS) and KRS-D1(qAC) (red), for 6 h followed by Hoechst stain (blue), and Z-stack was captured by a confocal microscope with a 63× objective. Three different 1.5 μm slices of the same cell are presented. Black and white images of selected cells from one stack are shown on the right. (c) Nuclear costain of probes was quantified by NIS-Element’s image acquisition software, quantifying images of several experiments, and overall, more than 40 cells per probe, data is normalized to costain of KRS-D1 probe determined as 100%.

Based on the positive biochemical results in the NIH-3T3 and HeLa cells, we initiated fluorescence imaging studies. Live monolayers of NIH-3T3 cells were treated with KRS probes for 6 h, followed by DNA staining with Hoechst dye, a short wash, and immediate imaging of the live cells by fluorescence microscopy. The results show intense staining of the probes containing CPP (KRS-A1(TAT), KRS-B1(qTAT), KRS-E1(qNLS) and KRS-F1(NLS)) and relatively weak staining of the probes lacking the CPP moiety (KRS-C1(AC) and KRS-D1(qAC)), (Figure 3a). The samples labeled with nonquenched probes had relatively higher backgrounds, although most were washed out before imaging.

We then imaged the fluorescently labeled cathepsins by using high-resolution microscopy. NIH-3T3 cells were incubated with KRS probes for 6 h and then stained with Hoechst before live microscopic analyses. Using a confocal microscope, we examined the colocalization of KRS probes with Hoechst to determine the spatial organization of cathepsins in the cell. To prevent false positive labeling beyond the geometric bounds of the nucleus, we performed multidimensional Z-stack analyses to scaffold the accurate three-dimensional image of the cell. Confocal Z-stack imaging reveals that CPP probes KRS-B1(qTAT) and KRS-E1(qNLS) label nuclear cathepsins in distinct slices of the same cell, as indicated by white arrows and costaining with Hoechst. In contrast, the control probe KRS-D1(qAC) did not detect cathepsins in the nucleus (Figure 3b). These data demonstrated that the enhanced permeability of the CPP probes enabled improved visualization of the active intracellular cathepsins and further demonstrated the presence of active cathepsins in the cell nucleus. The numerical quantification and analysis of the nuclear costain (Figure 3c) highlights the differences in the labeling of CPP probes versus the non-CPP probe, indicating the importance of the cell-penetrating peptides to the enhanced cellular and nuclear permeability.

#### Dynamics of KRS Probes in Live Cells

**Figure 4:**
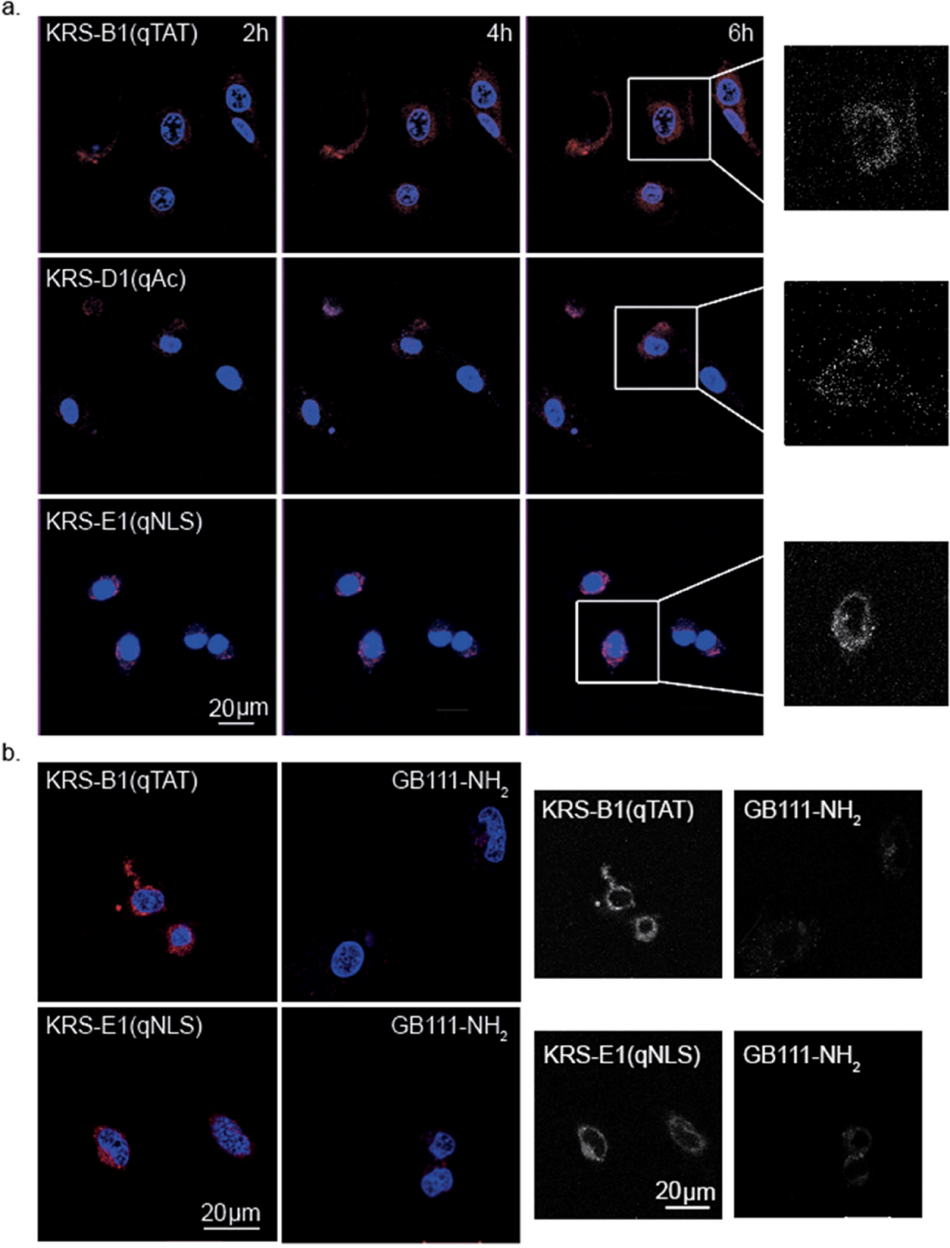
Dynamics of KRS probes in live cells by Fluorescent Confocal Microscope. (a) Intact monolayers of NIH-3T3 were labeled with 2.5 μM quenched probes KRS-B1(qTAT), KRS -E1(qNLS) and KRS-D1(qAC), and Hoechst stain for indicated times and captured with a Zeiss confocal microscope as in Figure 3a. Enlarged black-and-white fluorescent images of the probe signal from a single cell of the last time point are shown on the right. (b) Live HeLa cells were pretreated either with the 2.5 μM **GB111-NH**_**2**_ or with DMSO vehicle followed by labeling with probes KRS-B1(qTAT) or KRS-E1(qNLS), for 6 h, followed by Hoechst stain and captured by confocal microscopy. Red color, TMR-X fluorescence, blue color, Hoechst. Black-and-white fluorescent images of the probe signal are presented on the right.

Next, we observed the time-dependent activation and localization of cathepsins using KRS probes in live cells over extended time periods. Monolayers of NIH-3T3 were incubated with different KRS probes followed by imaging with a confocal microscope for 6 h with 2 h intervals (Figure 4a). Our data showed an increased fluorescence signal over time that peaked at ∼6 h. This is consistent with the time course experiments previously shown in (Figure 2). As demonstrated previously, CPP probes displayed an efficiency greater than that of the control probe KRS-D1(qAC) for cathepsin labeling. In addition, we found that KRS-B1(qTAT) and KRS-E1(qNLS) were mostly localized to the peri-nuclear region of the cell, while KRS-D1(qAC) was more scattered in the cell. Collectively, these data indicate the unique cellular localization of each probe.

Next, we tested the selectivity of KRS probes for the cathepsin enzymes in live cells. HeLa cells were labeled with KRS probes that were pretreated with **GB111-NH**_**2**_ or control vehicle for 1 h. Hoechst was then added, and the cells were visualized by confocal microscopy. The fluorescent signal was strongly reduced in samples pretreated with the cathepsin inhibitor **GB111-NH**_**2**_, demonstrating the high selectivity of the probes for the cathepsins (Figure 4b).

#### Monitoring Cathepsin Activity in Live NIH-3T3 and Fixed HeLa Cells

We next aimed to validate probe nuclear localization in a large population of cells. Using an Image Streamer device, we analyzed ∼50,000 cells per sample. We examined the colocalization of KRS probes, labeled with TMR-X and Hoechst for nuclei, focusing on the nuclear signal in different cell cycle stages.

**Figure 5:**
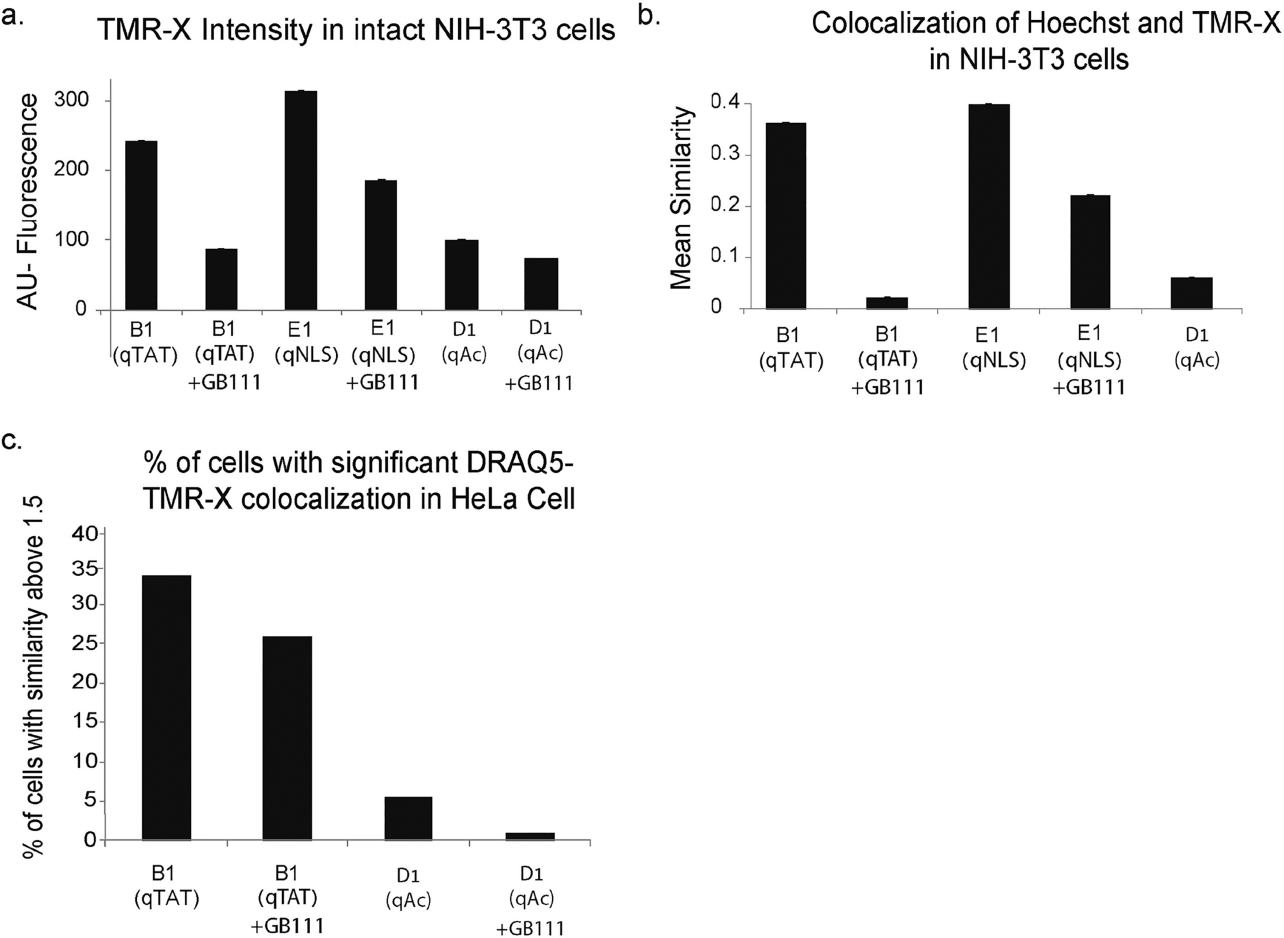
Analyses of probe localization in large populations of cells. Live NIH-3T3 cells were treated with 2.5 μM probes KRS-B1(qTAT), KRS -E1(qNLS), or KRS-D1(qAC) for 6 h with or without inhibitor pretreatment followed by Hoechst staining and analyzed by an Image Streamer device. Quantification of TMR-X intensity, data from approximately 50,000 cells. (a) TMR-X intensity of different probes in cells. (b) Mean similarity (colocalization) of the probe stain TMRX- and Hoechst. (c) HeLa cells were fixed, treated with probes B or D for 6 h with or without inhibitor pretreatment followed by Draq5 stain. The image of data was as received from Image Streamer. Statistical analysis of TMR-X and Draq5 similarity presents the presence of significant colocalization of the probe in the nucleus. These experiments were performed twice.

To this end, live NIH-3T3 were pretreated with either the cathepsin inhibitor **GB111-NH**_**2**_ or DMSO vehicle and then labeled for 6 h with KRS probes. After incubation, the cells were costained with Hoechst and Lyso-Tracker and analyzed by Image Streamer. As anticipated, we detected a higher fluorescent signal in the samples treated with CPP probes compared to KRS-D1(qAC) [control probe] (Figure 5a). In addition, the overlap between TMR-X signals (indicating active proteases) and the Hoechst (nuclear) was also increased in cells treated with CPP probes. This indicates that our probes are on target and suggests the presence of active cathepsins in the nucleus (Figure 5b).

To confirm that our data are general and not a consequence of a particular cell line nor a particular reagent, we repeated the experiments in HeLa cells using an orthogonal nuclear stain, Draq5, and KRS probes followed by cell fixation. Here too, we could show nuclear cathepsin activity both with the KRS-D1(qAC) probe lacking CPP and to a much higher degree with the KRS-B1(qTAT) probe (Figure 5c).

#### Evaluation of Cathepsin Nuclear Labeling Using Cell Fractionation

**Figure 6:**
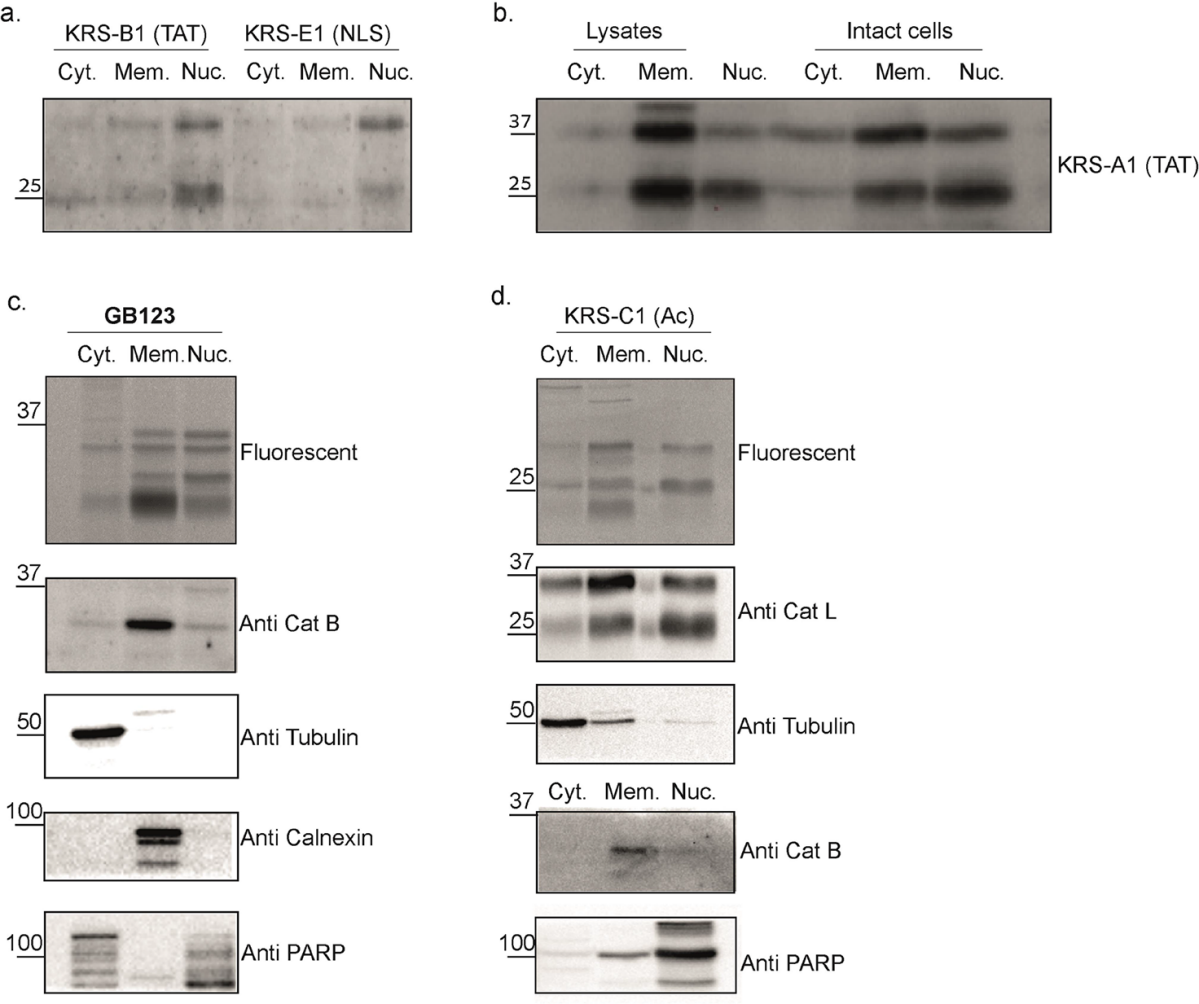
Cell fractionations show cathepsin activity in the nucleus. (a) HeLa cells were treated with 2.5 μM probes KRS-B1(qTAT) or KRS-E1(qNLS) for 6 h, lysates were fractionated for cytosolic fraction (Cyt.), membranes fraction (Mem.), and nuclear fraction (Nuc). Equal amount of protein samples, 20 μg, of each fraction were separated by SDS-PAGE and analyzed for fluorescent by a Typhoon laser scanner. (b) HeLa cells were either first lysed then treated with KRS-A1(TAT) for 6 h or labeled with KRS-A1(TAT) and then lysed. Lysates were fractioned to Cyt., Mem., and Nuc. extracts. The extracts were separated by SDS-PAGE and blotted with anticathepsin L antibody. (c) HeLa cells were treated with 1 μM GB123 (a Cy5 nonquenched probe) for 6 h then lysed, fractionation was preformed similarly and were separated by SDS-PAGE and analyzed for fluorescent by a Typhoon laser scanner. The gel was then blotted and reacted with Cath B, Tubulin, calnexin, and PARP antibodies. (d) HeLa cells were treated with 2.5 μM KRS-C1(Ac) for 6 h then lysed and analyzed as in C with indicated antibodies.

Next, we sought to validate our imaging and biochemical results using subcellular fractionation. Cell fractionation was validated (Figure S3). HeLa cells were labeled with KRS-B1(qTAT) or KRS-E1(qNLS) probes and then lysed in a manner that recovers subcellular organelles. The fractions were separated by SDS-PAGE and cathepsin activity was measured on a gel. In concordance with our imaging data, nuclear fractions of the sample treated with KRS-B1(qTAT) and KRS-E1(qNLS) demonstrated a clear presence of active nuclear cathepsin (Figure 6a). KRS-E1(qNLS) showed the same trend as KRS-B1(qTAT) but had a weaker intensity. This further demonstrates the ability of CPP groups to target the nucleus and fully supports our imaging results.

Next, we investigated whether the KRS-TAT probe labels nuclear cathepsins in situ or whether this probe is in fact mediating the lysosomal-nucleus transition of cathepsins; we performed parallel labeling of two samples. In the first sample, we labeled cells with KRS-A1(TAT) probe followed by cell lysis and fractionation, and in the second, we first lysed the cells, then labeled the lysates with KRS-A1(TAT) followed by fractionation. We selected the TAT containing KRS-A1(TAT) probe for this experiment since it showed the strongest binding of all probes as seen in (Figures 2 and 6a). The fractions of both samples were analyzed by SDS-PAGE and blotting with an anticathepsin L antibody. We found more nuclear cathepsin L after labeling with the probe in intact cells compared to the nuclear cathepsin L after lysate labeling (using equal amounts of proteins for each sample); importantly, a significant amount of nuclear cathepsin L was detected in the nucleus of cells that were first lysed (Figure 6b). Therefore, we concluded that nuclear localization of cathepsins exists naturally in cells and there is an additional amount that is driven to the nucleus because of the TAT-probe attachment.

To further determine the physiological levels of nuclear active cathepsins, we treated cells with cathepsin probes lacking a CPP and analyzed the different cell fractions: cytosolic, membrane, and nuclear fractions. We used KRS-C1(AC) and our published **GB123** that both target multiple cathepsins and lack a CPP moiety.^34^ The fractions were first analyzed by fluorescent SDS page, and then the gels were blotted and reacted with cathepsin antibodies. Lastly, the samples were rerun and reacted with antibodies to other cathepsins and proteins that are known to localize to specific cell compartments to confirm the quality of the fractionation (Figure 6c,d). These results showed that cathepsin L is highly active in the nucleus while cathepsin B is less and that the fractionation was adequate. This experiment further confirms that the nuclear cathepsin L activity is independent of the probe’s CPP.

#### Nuclear Cathepsin Activity Changes during Cell Division

**Figure 7:**
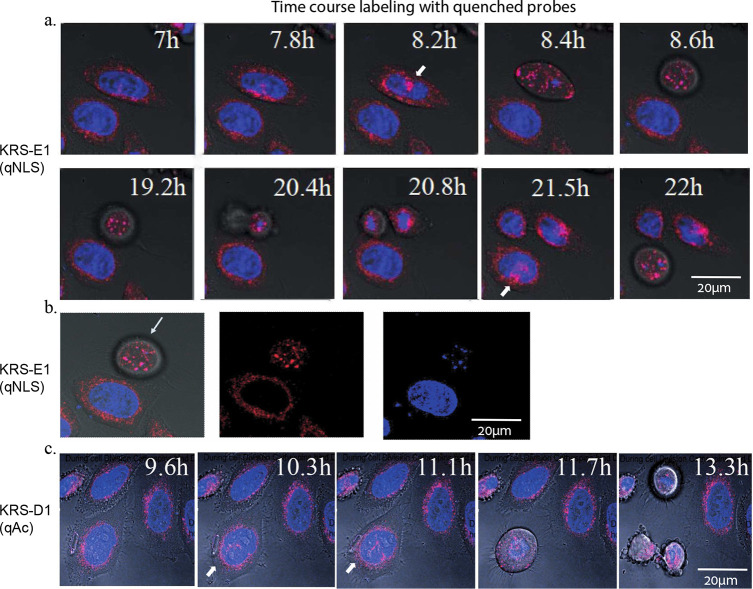
Nuclear cathepsin appears in accordance with cell cycle HeLa cells were labeled with 2.5 μM KRS probes and stained with Hoechst, followed by immediate confocal microscopic analyses, pictures were taken every 20–25 min over 22 h and were used to generate time lapse movies. Selected images from the time laps move are presented, Cy3 (probe fluorescence) in red and Hoechst (DNA) in blue. The time printed in white character indicates the hours after probe addition. (a) Images from KRS-E1 (qNLS) labeling are presented, the movie is presented in Movie S1. A white arrow indicates an individual cell prior to mitosis with nuclear cathepsin activity. (b) Selected image from KRS-E1 (qNLS) at 8.5 h after probe addition. On the left an overlay of Cy3 (probe) and Hoechst (DNA stain), in the middle of the Cy3 signal, and on the Hoechst signal. (c) HeLa cells were treated with KRS-D1(qAc) probe lacking a CPP moiety, similarly, (images from Movie S2) nuclear cathepsins are localized to the nucleus prior to mitosis indicated by white arrow.

A few reports suggest that nuclear localization of cathepsins is time-dependent and occurs at specific time points during cell cycle progression.^35,22,7^ Thus, HeLa cells were treated with KRS-D1(qAC), KRS-B1(qTAT), or KRS-E1(qNLS) together with Hoechst, and fluorescence images of the live cells were captured every 20–25 min over 22 h, generating time-lapse movies (Movies S1, S2, S3). We were able to detect cathepsin activity in live cells even after 22 h. Pictures from the time-lapse movie of KRS-E1(qNLS), (Movie S1), are presented in (Figure 7a). Over the hours the movies were captured, several cells entered mitosis; this was observed by rounding off the cells and chromosome condensation, followed by the generation of two daughter cells. The data show a remarkable phenomenon that repeats itself and is easily revealed using the chemical tools described in this manuscript. During most of the cell cycle, the most active cathepsins occupy the lysosome. However, just before mitosis, a large amount of cysteine cathepsins localizes to the nucleus. The probe in the nucleus was found to be colocalized with the DNA as shown in (Figure 7b). This phenomenon was also detected when cells were treated with KRS-D1(qAC) (lacking a CPP), (Figure 7c), (Movie S2). Surprisingly, treatment with KRS-B1(qTAT) inhibited the entry of cells to mitosis (Movie S3), since a large amount of probe-labeled cathepsins enter the nucleus, it likely impairs this highly regulated process of mitosis as the cathepsin activity is inhibited when binding the probe.

## Discussion and Conclusions

Cysteine cathepsins play key roles in intracellular protein turnover and are also recognized as key players in tumor progression and other pathologies. Recent reports show that cathepsins localize to the cell nucleus and not only to the lysosome.^36^ Few studies have identified biological roles for nuclear cathepsins during cell cycle progression and in DNA transcriptional and translational regulation^9,37^ In our effort to study the dynamic nuclear cathepsin function we present the design and application of novel chemical tools, namely, quenched activity-based probes conjugated to cell-penetrating peptides (CPP). These probes are ideal to study changes that occur over short periods of time that could be detected using real-time imaging.

Using recombinant cathepsins B, L, and S, we found that all KRS probes specifically bind cathepsins in a dose-dependent manner. We further noticed that larger probes such as KRS-B1(qTAT) and KRS-E1(qNLS) had lower affinity for the enzymes compared with smaller molecules such as KRS-D1(qAc) and KRS-C1(Ac). The decrease in the affinity was likely due to steric hindrance of the large probes, which interfered with the space-restricted catalytic pocket of the enzymes (Figure 1). Nevertheless, labeling of the endogenous cathepsins in intact cells showed the opposite trend. The large CPP probes, despite having lower affinity in enzyme-labeling experiments, demonstrated more efficient labeling of endogenous enzymes. Specifically, the TAT- and NLS-containing probes, KRS-B1(qTAT) and KRS-E1(qNLS), showed significantly greater cell penetration than the control probe KRS-D1(qAc) (Figure 2). The increase in the fluorescent signal of the labeled cathepsins with CPP-probes was also demonstrated during the confocal microscopy studies fitting to the biochemical data (Figure 3a). Therefore, the CPP group efficiently enhances the cell permeability, overcoming the decreased affinity drawback. The decrease in labeling of the samples treated with the cathepsin inhibitor indicates the high selectivity of the KRS probes, also demonstrated in microscopy assays (Figure 4b). We also noticed that the quencher group decreases the cell permeability while comparing all the quenched probes to their nonquenched analogs (Figure 2).

Cell fractionation in HeLa cells showed that the majority of nuclear cathepsins labeled with our probes were in fact cathepsin L and not cathepsin B. While the CPP enhances nuclear entry similar phenomena of nuclear cathepsins were detected also with ABPs lacking a CPP (Figures 6 and 7). Most importantly, we performed cellular assays that demonstrated temporal cathepsin nuclear entry. While during most of the cell cycle low levels of active cathepsins were detected in the nucleus, we detected a surge of nuclear cathepsins immediately before mitosis, (Figure 7).

Our finding of timely entry to the nucleus implies that cathepsins have specific nuclear functions that are linked to cell cycle progression. This fits well with our previous finding that cathepsin L knock-out (KO) MEF cells proliferate faster than both wild-type cells and cathepsin B KO cells.^38^ However, due to the very high similarity and known nuclear localization, we cannot exclude that our probes also labeled cathepsin V. Nevertheless, in addition to the known cathepsin functions, future search for additional nuclear cathepsin substrates will surely shed light on how cathepsins are involved in the cell cycle.

Our data raise several questions, first and foremost: how does cathepsin L enter the nucleus in a cell cycle-dependent manner? We assume that Cathepsin L shuttling to the nucleus is achieved by binding to proteins or complexes that migrate to the nucleus in a cell cycle-dependent manner. On these lines, we propose two possible mechanisms: cathepsin L was reported to bind to Snail, a transcription factor that contains an NLS, in the cytoplasm. Snail migrates to the nucleus, after binding to importin β.^39^ The regulation of Snail’s nuclear entry is done by Pak1 phosphorylation in a cell cycle manner since Pak1 was shown to be active in the cytoplasm in both G2/M as well as before mitosis.^40^ Thus, a cathepsin L- Snail complex could be shuttled to the nucleus in a cell cycle-dependent manner.^41^ Furthermore, cathepsin L nuclear accumulation could be achieved by changing the balance between nuclear export and import, specifically blocking nuclear export and resulting in nuclear accumulation. Indeed, the Schilling lab reported that cathepsin L cleaves Exportin 1,2,5 and Exportin T, using proteomic identification of protease cleavage sites (PICS), Exportin cleavage should lead to nuclear accumulation.^42^

The mechanism of entry of TAT into cells remains unclear. While several studies favor the endocytic mechanism,^43−45^ entry of TAT probes through the endocytic pathway could explain our findings with KRS-B1(qTAT) (Movie S3) and KRS-A1(TAT) (Figure 6b), where the probe would arrive at the lysosome through endocytosis, and cause extensive lysosomal cathepsin labeling. In the next step, this complex migrates to the nucleus.

Although a direct link between nuclear cathepsin activity and cell mitosis was not previously shown, several reports suggest evidence of this effect. Terayama et al. showed that cathepsin L injected intraperitoneally into mice stimulated DNA synthesis and mitosis in the intact and cultured liver.^46^ Another finding by Sylvén et al. showed that the cell surface of tumor cells has increased cathepsin B during premitotic and mitotic phases.^47^ One of the few known nuclear substrates of cathepsin L is histone H3 which undergoes phosphorylation during cell division.^15,47,48^ Based on this evidence, we speculate that one of the possible cathepsin functions in the cell nucleus might be promotion of the cell proliferation via chromatin remodeling with H3 as a specific target.

In conclusion, we demonstrate the design and synthesis of novel, specific, and selective qABPs for labeling cathepsins. The CPP attachment was utilized for enhanced cell permeability and nuclear cathepsin detection. The concept was successfully proved in two cell lines, NIH-3T3 and HeLa using different approaches, such as biochemistry and imaging. As a result, the array of novel probes that were developed enables real-time tracking of nuclear cathepsins. These probes allow for multifaceted investigation and study of cathepsin involvement in the cell cycle. New findings based on the use of these probes may prove valuable for developing new strategies for therapeutic approaches and understanding the physiological mechanisms of various diseases.

## Supporting Information Available

Quantifications of the interaction between cellular and nuclear cathepsin with probes; labeling of endogenous cathepsins in intact WT and cathepsin knockout cells; cathepsin targets of KRS-A1 (TAT) and KRS-B1 (qTAT) probes in intact cells; and validation of sub-cellular fractionation in Hela cells (PDF)
Time-lapse movie of HeLa cells treated with KRS-E1(qNLS) over 22 (Movie S1) (AVI)
Time-lapse movie of HeLa cells treated with KRS-D1(qAC) (lacking a CPP) (Movie S2) (AVI)
Time-lapse movie of HeLa cells treated with KRS-B1(qTAT) inhibiting the entry of cells to mitosis (Movie S3) (MP4)

## Supplementary Material

se4c03217_si_001.pdf

se4c03217_si_002.avi

se4c03217_si_003.avi
