# Supplementary material for: Novel Nucleus-Oriented Quenched Activity-Based Probes Link Cathepsin Nuclear Localization with Mitosis
Source: ACS Sens. 2025 Feb 17;10(2):1321–33. doi: 10.1021/acssensors.4c03217 (PMC11877631; doi:10.1021/acssensors.4c03217)
Supplement: Supplementary file 1 — se4c03217_si_001.pdf [file se4c03217_si_001.pdf]

Supporting Information

Novel Nucleus-Oriented Quenched Activity-Based Probes Link Cathepsin Nuclear Localization with Mitosis

Karin Reut Shannon,[1] Tommy Weiss-Sadan,[1] Emmanuelle Merquiol,[1] Gourab Dey,[1] Tamar Gilon,[2] Boris Turk, [3,4] and Galia Blum[*1,5]

1. The Institute for Drug Research, The School of Pharmacy, The Faculty of Medicine, The Hebrew University, Jerusalem, Israel.

2. Azrieli College of Engineering, Jerusalem, Israel,

3. Department of Biochemistry and Molecular Biology, J. Stefan Institute, Ljubljana, Slovenia.

4. Faculty of Chemistry and Chemical Technology, University of Ljubljana, Slovenia.

5. The Wohl Institute for Translational Medicine, Hadassah Hospital, Jerusalem, Israel

[*]Corresponding author

Galia Blum, Email: galiabl@ekmd.huji.ac.il

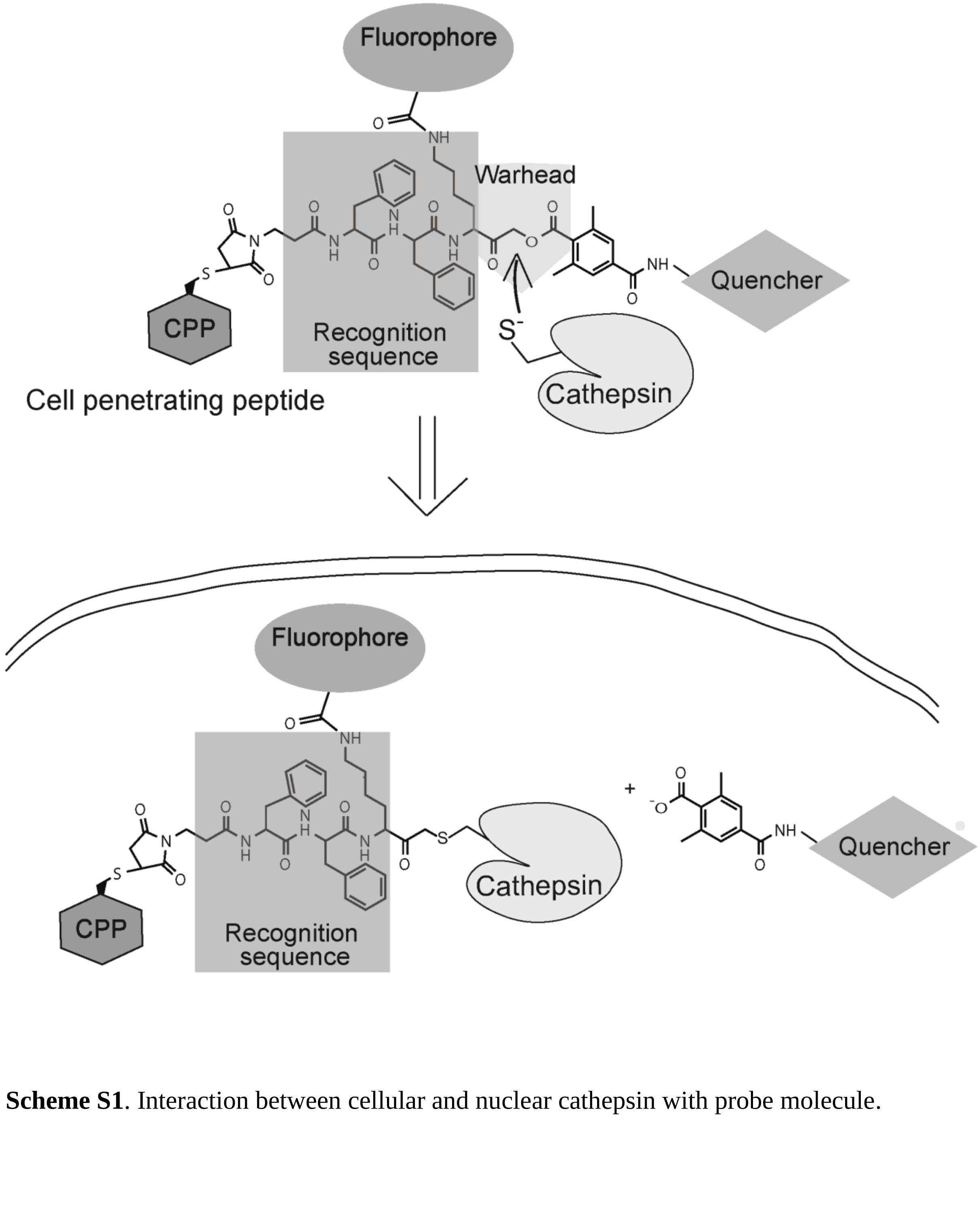

Scheme S1. Interaction between cellular and nuclear cathepsin with probe molecule.

Labeling of endogenous cathepsin in intact cells

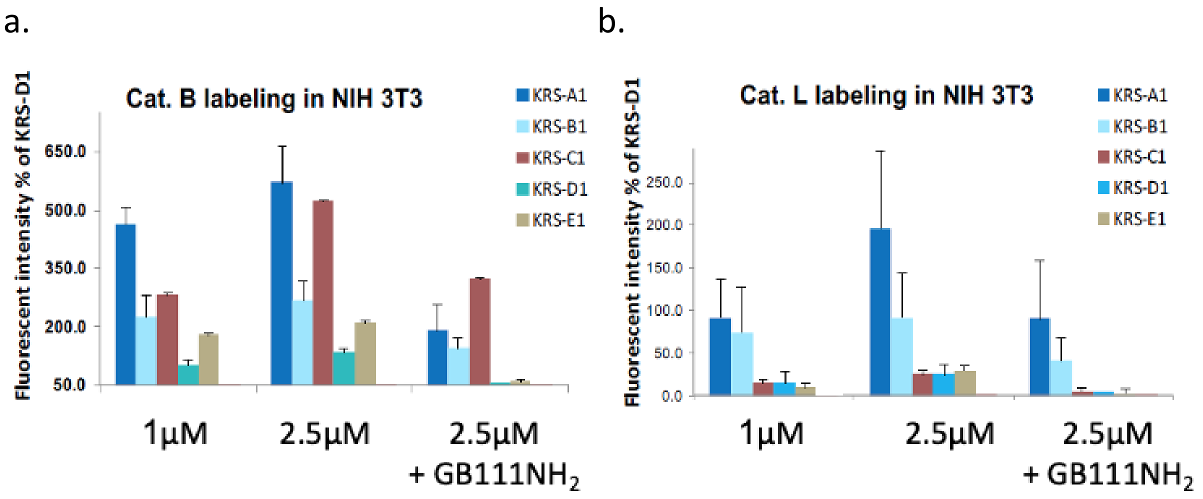

Figure S1. Labeling of endougenous cathepsins in intact cells. Intact monolayers of NIH-3T3 were pretreated either with the GB111-NH_2 or with DMSO vehicle followed by labeling with probes at the indicated concentration. Cell lysates were collected, separated by SDS-PAGE and analyzed for Cy3 fluorescent by a Typhoon laser scanner. This experiment was repeated more than three times with similar results. Plots show the quantification average of band intensity of **(a)** cathepsin B labeling and **(b)** cathepsin L labeling, using Image-J program with standard deviation.

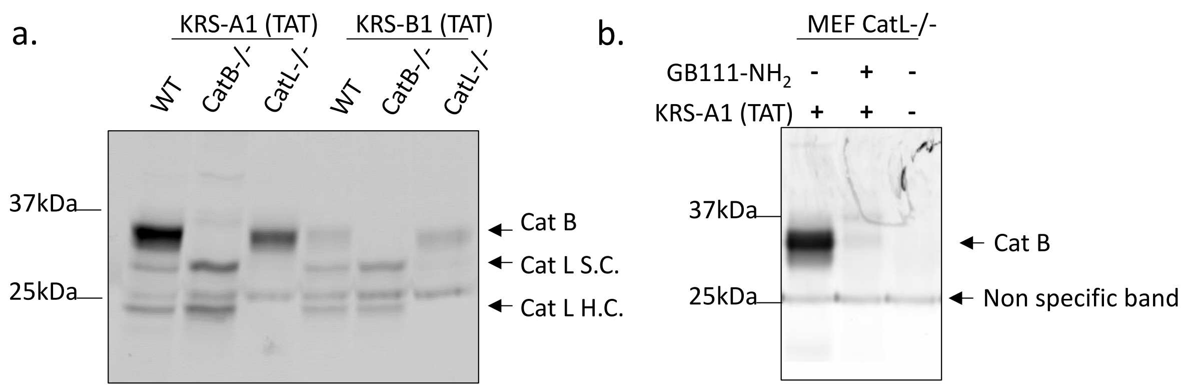

Figure S2. Cathepsin targets of KRS-A1 (TAT) and KRS-B1 (qTAT) probes in intact cells**.** (a) Intact monolayers of mouse embryonic fibroblasts (MEF), wild type, cathepsin B-/- and cathepsin L-/- were pre-treated either with 5 μM GB111-NH2 or with DMSO vehicle for one hour, followed by labeling with 1 μM KRS-A1 (TAT) or 2.5 μM KRS-B1 (qTAT) for 8 hours. Cell lysates were with collected, and equal protein of each sample were separated by SDS-PAGE and analyzed for Cy3 fluorescent by a Typhoon laser scanner. Naïve cells were used as controls .The experiment was repeated twice with similar results. These probes bind cathepsin B and two isoforms of cathepsin L the single chain (S.C.) and the heavy chain (H.C.)

marked by arrow. * marks a non-specific band present in cells (see b.) (b) Intact cathepsin L-/- were treated similar to as described in a. KRS-A1 (TAT) or with DMSO vehicle. A non-specific band above 25kDa was detected in vehicle-treated cells without a probe.

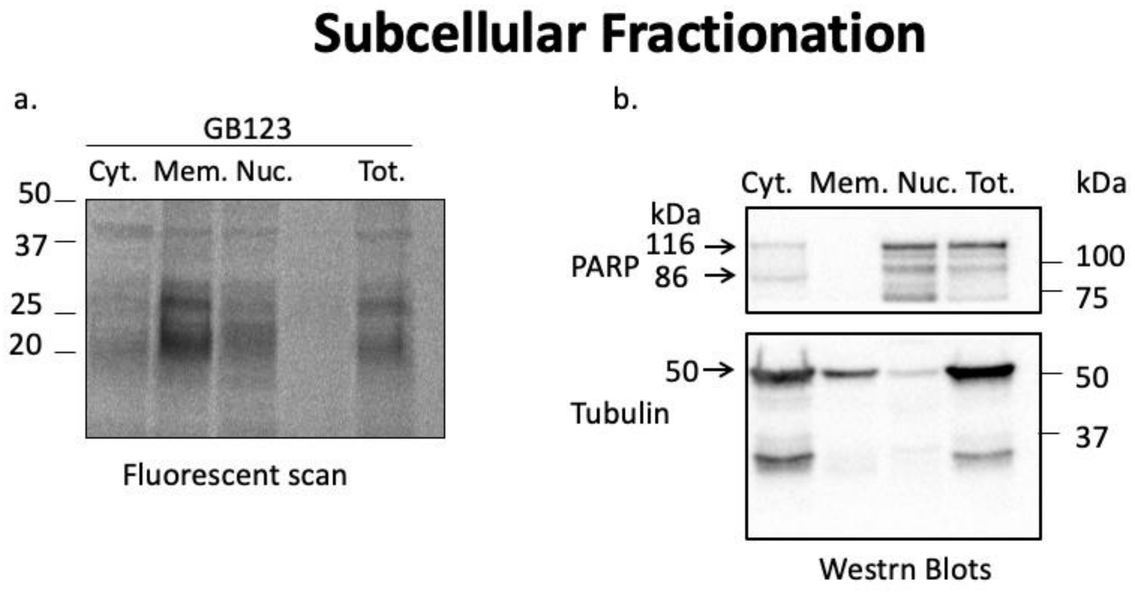

Figure S3. Valiadation of sub-cellular fractionation in Hela cells. (a) Hela cells were treated with 1 µM GB123, (a Cy5 non-quenched probe) for 6 h, lysates were fractionated for cytosolic fraction (Cyt.), membranes fraction (Mem.), and nuclear fraction (Nuc), total (Tot.) cell lysate was run as control. The fractions were separated by SDS-PAGE and analyzed for fluorescent by a Typhoon laser scanner. (b) Hela cells were treated with 1 µM GB123 (a Cy5 non-quenched probe) for 6 h then lysed, fractionation was performed similarly and were separated by SDS-PAGE. The gel was then blotted and reacted with anti PARP and anti Tubulin, antibodies as markers of the nuclear fraction and the cytosolic fraction.
